# Synthesis, crystal structure and Hirshfeld surface analysis of 2-phenyl-3-(prop-2-yn-1-yl­oxy)quin­oxaline

**DOI:** 10.1107/S2056989024002585

**Published:** 2024-03-21

**Authors:** Nadeem Abad, Joel T. Mague, Abdulsalam Alsubari, El Mokhtar Essassi, Mehrdad Pourayoubi, Abdullah Yahya Abdullah Alzahrani, Youssef Ramli

**Affiliations:** a Laboratory of Medicinal Chemistry, Drug Sciences Research Center, Faculty of Medicine and Pharmacy, Mohammed V University in Rabat, Morocco; b Laboratory of Heterocyclic Organic Chemistry, Faculty of Sciences, Mohammed V University, Rabat, Morocco; c Department of Chemistry, Tulane University, New Orleans, LA 70118, USA; d Laboratory of Medicinal Chemistry, Faculty of Clinical Pharmacy, 21 September University, Yemen; e Department of Chemistry, Faculty of Science, Ferdowsi University of Mashhad, Mashhad, Iran; f Department of Chemistry, Faculty of Science and Arts, King Khalid University, Mohail Assir, Saudi Arabia; g Mohammed VI Center for Research and Innovation (CM6), Rabat 10000, Morocco; Katholieke Universiteit Leuven, Belgium

**Keywords:** crystal structure, quinoxaline, alkyl­ation, hydrogen bond, π-stacking

## Abstract

In the title compound, C_17_H_12_N_2_O, the quinoxaline moiety shows deviations of 0.0288 (7) to −0.0370 (7) Å from the mean plane (r.m.s. deviation of fitted atoms = 0.0223 Å). In the crystal, corrugated layers two mol­ecules thick are formed by C—H⋯N hydrogen bonds and π-stacking inter­actions.

## Abstract 2

In the title compound, the quinoxaline moiety shows deviations of 0.0288 (7) to −0.0370 (7) Å from the mean plane (r.m.s. deviation of fitted atoms = 0.0223 Å). In the crystal, corrugated layers two mol­ecules thick are formed by C—H⋯N hydrogen bonds and π-stacking inter­actions.

## 1. Chemical context

**Figure 1:**
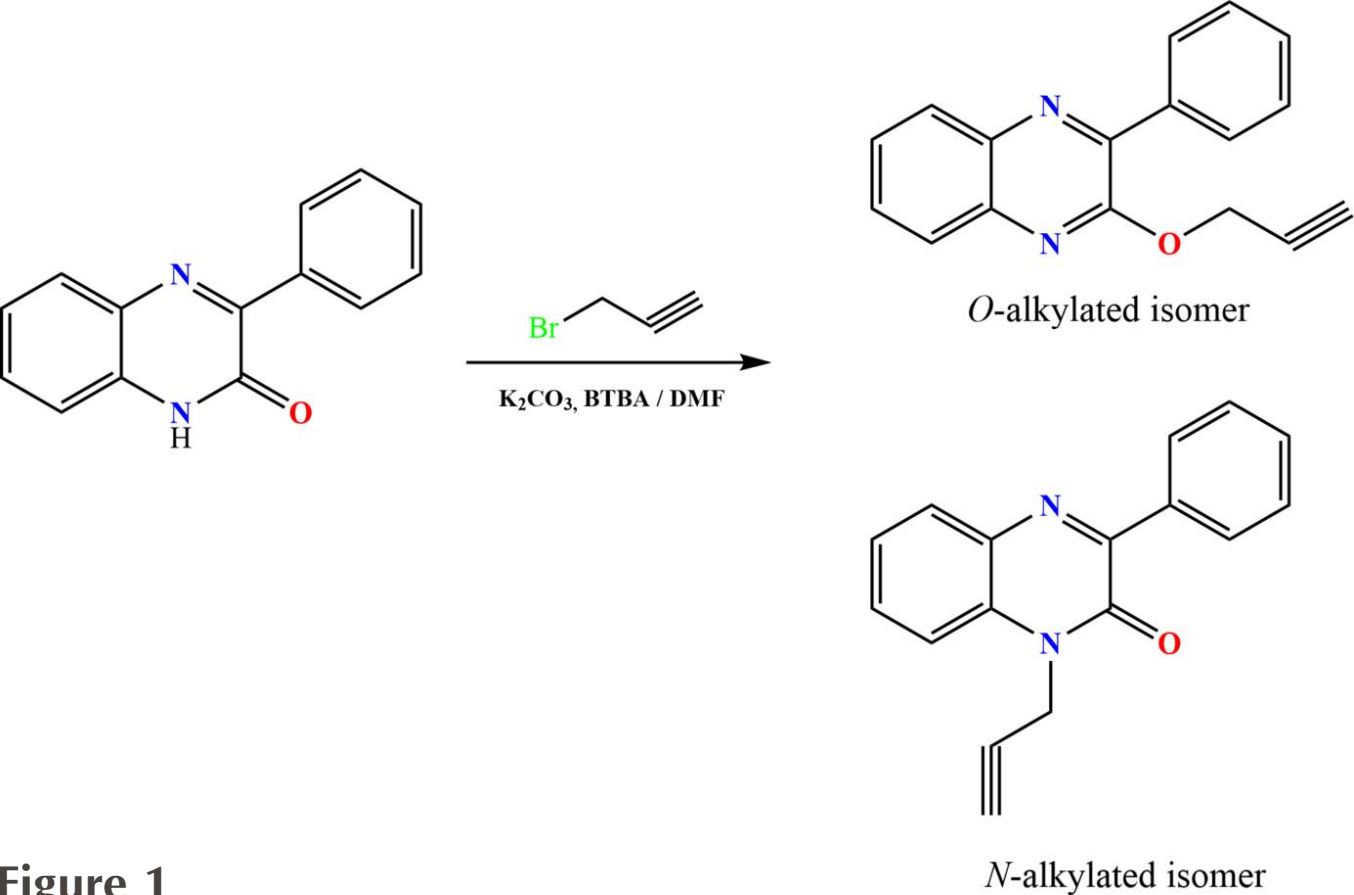
Synthesis of the title compound.

Quinoxaline derivatives are described extensively among the heterocycles being investigated for the discovery and development of new biologically active mol­ecules. Numerous studies have been published regarding this class of compounds, revealing that quinoxaline is present in a number of well-established drugs with diverse therapeutic activities as well as industrial properties (*e.g.* Lgaz *et al.*, 2015 ▸). In recent decades, the medicinal chemistry of quinoxaline and its deriv­atives have received great attention due to their wide spectrum of biological activities, in particular analgesic, anti-diabetic, anti­viral, anti­bacterial, anti­oxidant, anti-inflammatory, anti­depressant, and anti-tubercular (Ramli & Essassi, 2015 ▸). Our inter­est in quinoxalines results from their simple synthesis and the ease with which X-ray quality crystals can be grown. Following this line of research, and as a continuation of our work in this area (*e.g.* Missioui *et al.*, 2022 ▸), based on the therapeutic significance of this scaffold for potential applications in medicinal chemistry, we report herein the synthesis of a new quinoxaline derivative by an alkyl­ation reaction of 3-phenyl­quinoxalin-2(1*H*)-one using 3-bromo­prop-1-yne as an alkyl­ating reagent and potassium carbonate in the presence of tetra-*n*-butyl­ammonium bromide as catalyst in phase-transfer catalysis (Fig. 1 ▸). A Hirshfeld surface analysis was performed to analyze the inter­molecular inter­actions. 
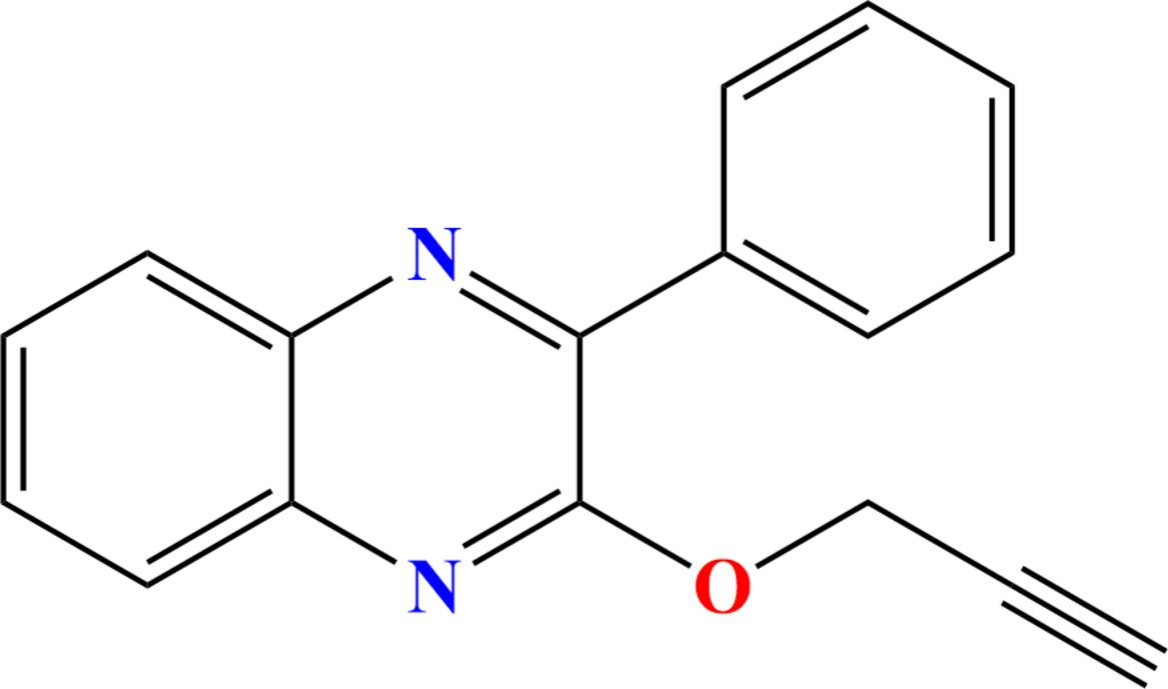


## 2. Structural commentary

**Figure 2:**
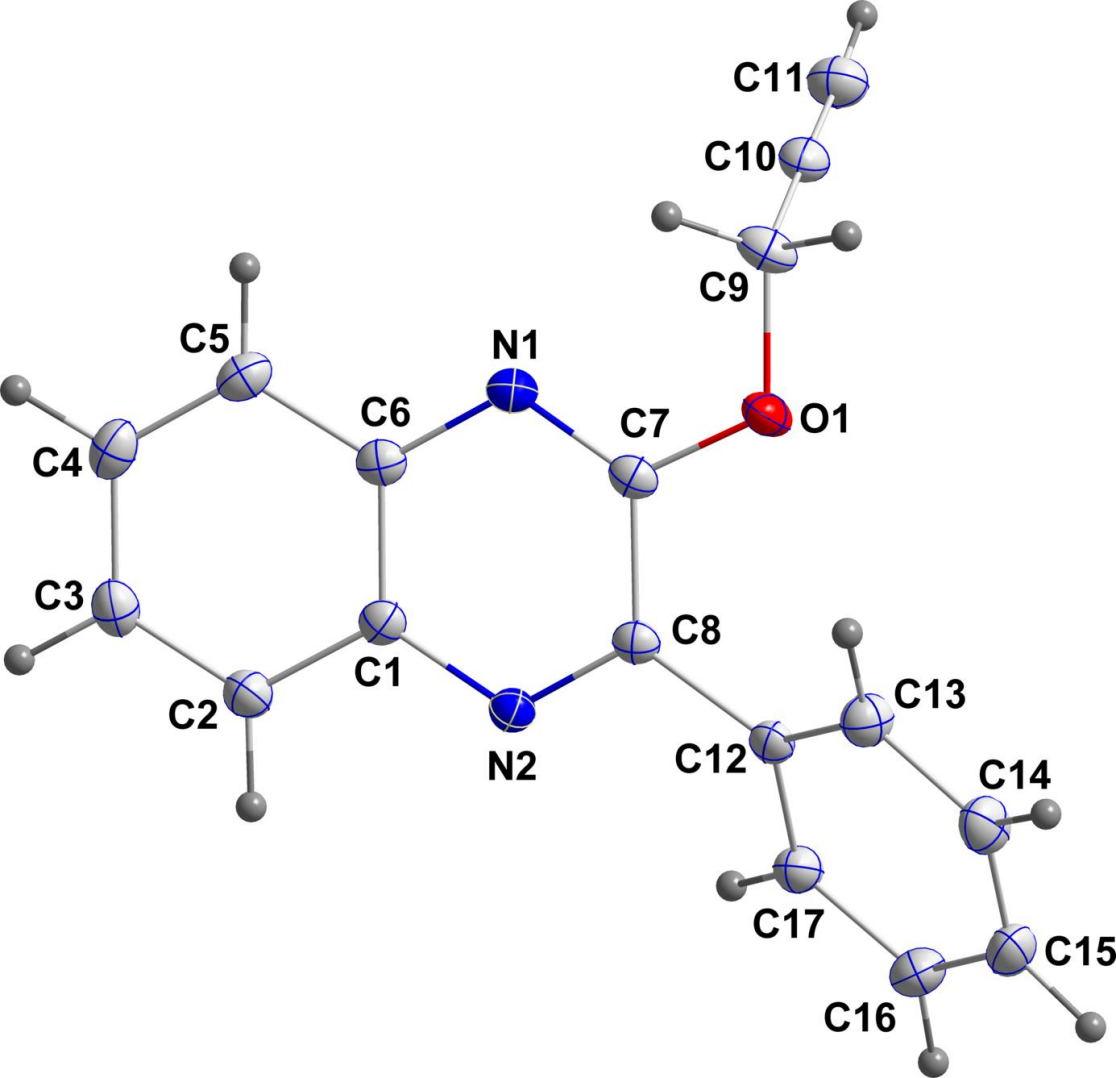
Mol­ecular structure of the title mol­ecule with labeling scheme and 50% probability ellipsoids.

In the title mol­ecule, the fused bicyclic ring system is not entirely planar, as indicated by the dihedral angle of 2.25 (6)° between its constituent rings and by the deviations from the mean plane through the ten atoms, which range from 0.0288 (7) Å (C8) to −0.0370 (7) Å (C2) (r.m.s. deviation of fitted atoms = 0.0223 Å). The plane of the benzene ring C12–C17 is inclined to the above plane by 34.04 (4)°, while the methyl­ene carbon of the propynyl group (C9) lies virtually in the plane of the quinoxaline unit, as indicated by the C9—O1—C7—N1 torsion angle of 0.65 (13)°. However, the propynyl group is almost perpendicular to the above plane, as indicated by the C7—O1—C9—C10 torsion angle of −87.0 (1)° (Fig. 2 ▸).

## 3. Supra­molecular features

**Figure 3:**
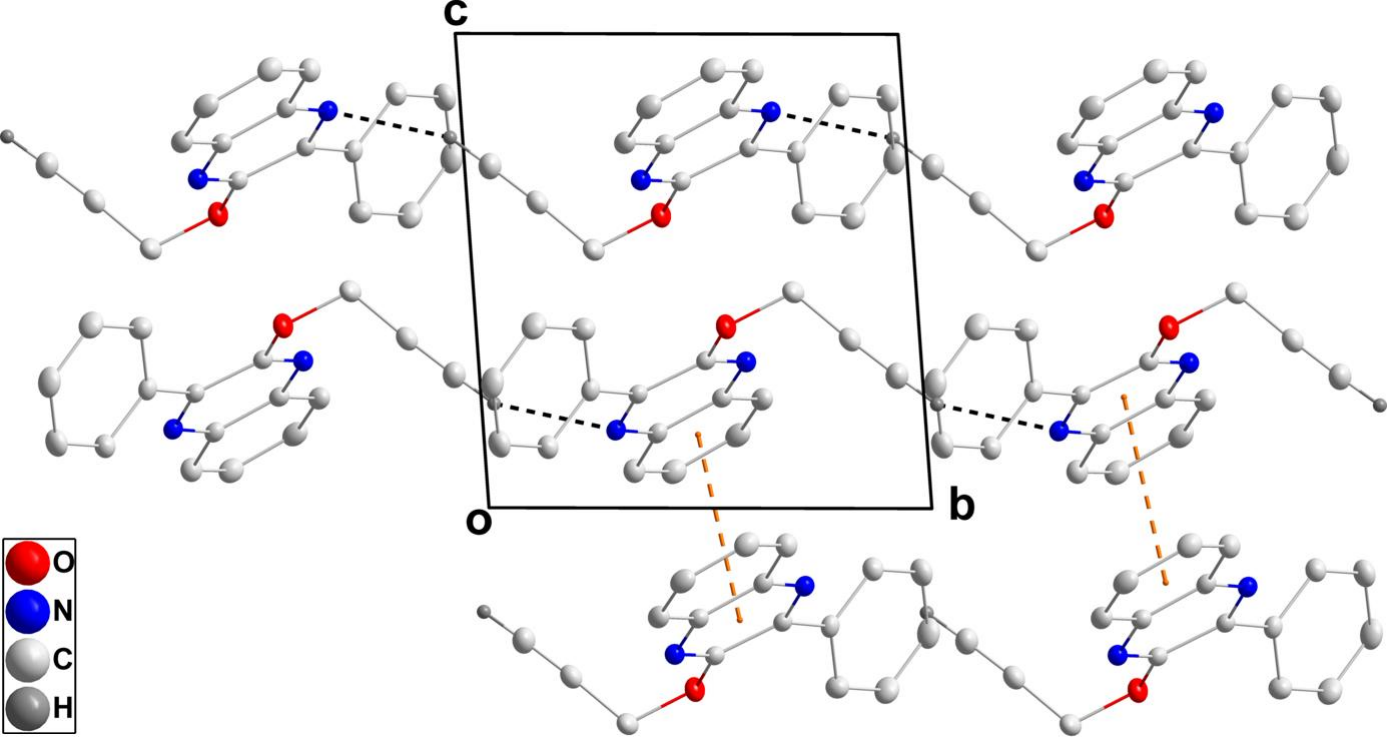
Packing viewed along the *a*-axis direction with C—H⋯N hydrogen bonds and π-stacking inter­actions shown, respectively, by black and orange dashed lines. Non-inter­acting hydrogen atoms are omitted for clarity.

In the crystal, the mol­ecules are connected into chains extending along the *b*-axis direction by C11—H11⋯N2 hydrogen bonds (Table 1 ▸ and Fig. 3 ▸). The chains are linked into corrugated layers two mol­ecules thick by offset π-stacking inter­actions between the C1–C6 and C1/C6/N1/C7/C8/N2 rings [centroid–centroid distance = 3.6716 (8) Å; dihedral angle = 2.25 (4)°, slippage = 1.262 Å] across inversion centers (Fig. 3 ▸).

**Figure 4:**
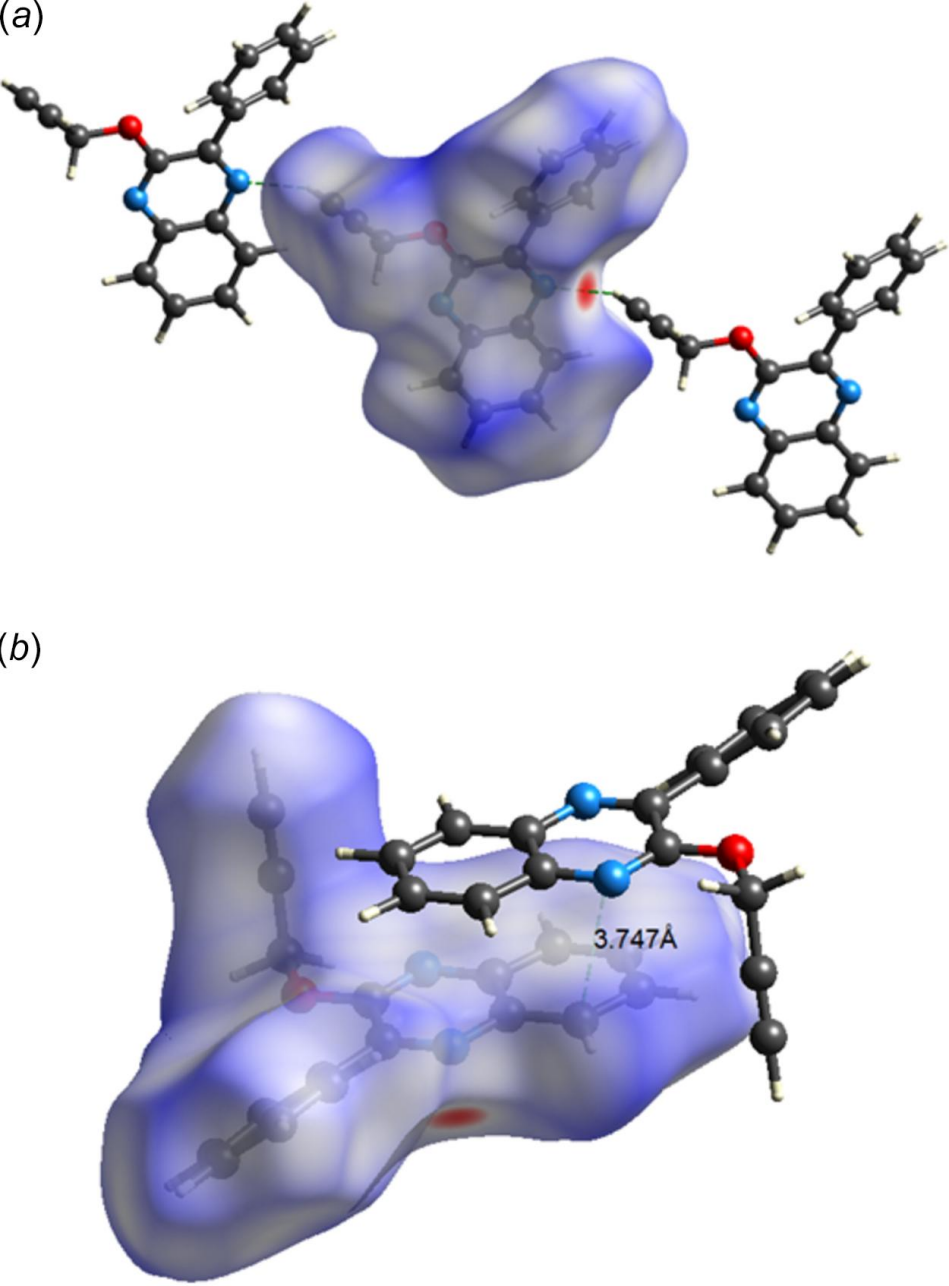
The Hirshfeld surface plotted over *d*_norm_ in the range −0.2356 to 1.4819 in arbitrary units) with (*a*) two neighboring hydrogen bonded mol­ecules and (*b*) one neighboring π-stacked mol­ecule.

**Figure 5:**
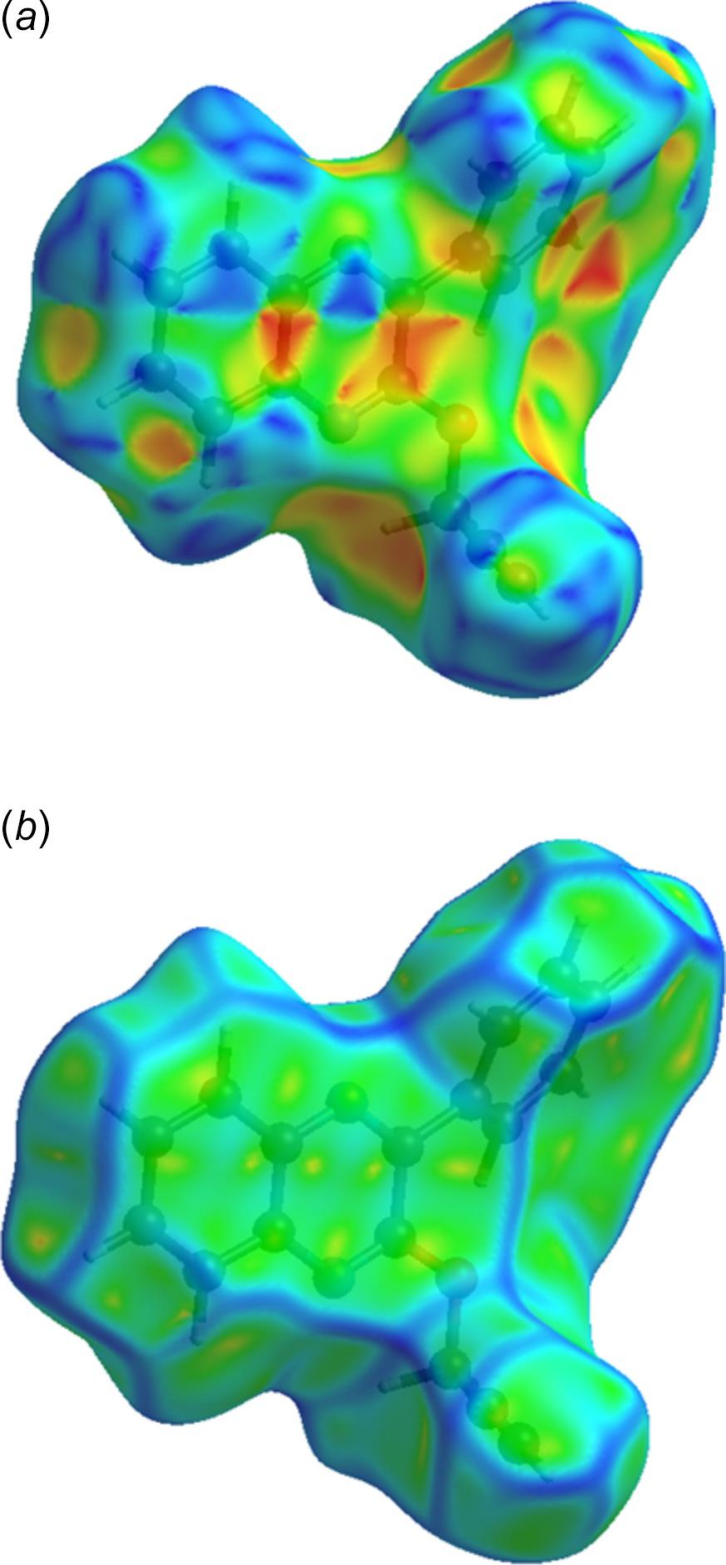
The Hirshfeld surface plotted over (*a*) shape-index and (*b*) curvature.

**Figure 6:**
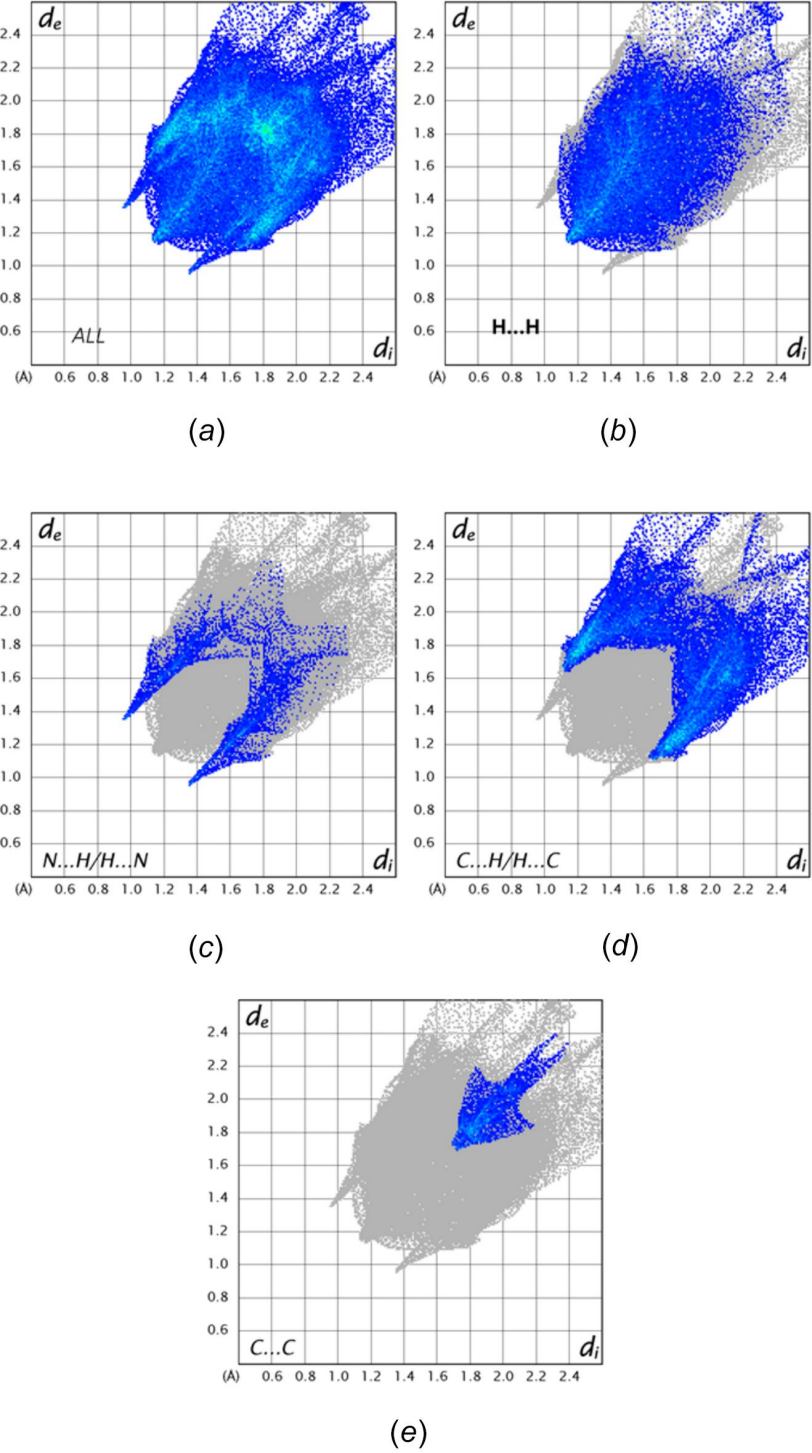
2D fingerprint plots for (*a*) all inter­molecular inter­actions, and delineated into (*b*) H⋯H, (*c*) N⋯H/H⋯N, (*d*) C⋯H/H⋯C and (*e*) C⋯C inter­actions.

To qu­antify the extent of each type of inter­molecular inter­action in the crystal packing, a Hirshfeld surface analysis was performed using *CrystalExplorer* (Version 21.5; Spackman *et al.*, 2021 ▸). Descriptions of the surfaces generated and their inter­pretation have been published (Tan *et al.*, 2019 ▸). Fig. 4 ▸ shows the *d*_norm_ surface with Fig. 4 ▸ *a* showing two neighboring mol­ecules illustrating the C—H⋯N hydrogen bond and Fig. 4 ▸ *b* one neighbor illustrating the π-stacking. From Fig. 4 ▸ *a*, it is clear that the C—H⋯N hydrogen bond is the only inter­molecular hydrogen bond in the structure. Fig. 5 ▸ *a* shows the surface mapped over shape-index while Fig. 5 ▸ *b* shows it mapped over curvature. In both of these, the characteristic features of inter­molecular π-stacking inter­actions are quite evident. Fig. 6 ▸ presents the 2D fingerprint plots with Fig. 6 ▸ *a* giving the total of all inter­molecular inter­actions and Fig. 6 ▸ *b*–6e showing those delineated into H⋯H, C⋯H/H⋯C, N⋯H/H⋯N and C⋯C inter­actions. These are the major inter­actions and contribute 42.8%, 36.8%, 8.3% and 6.3% to the total, respectively. In the absence of C—H⋯π(ring) inter­actions, the large contribution of C⋯H/H⋯C inter­actions may seem unusual, but *PLATON* (Spek, 2020 ▸) indicates that there are at least six and as many as ten such contacts with distances slightly shorter than to slightly longer than the sum of the respective van der Waals radii.

## 4. Database survey

**Figure 7:**
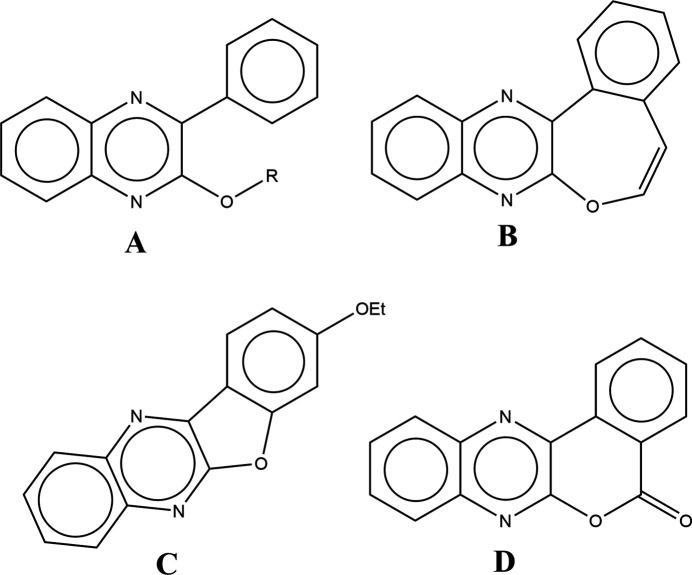
Search fragment (**A**), BZOQUX10 (**B**), VAQNAE (**C**) and YEFDUK (**D**).

A search of the Cambridge Structural Database (CSD, updated to January 2024; Groom *et al.*, 2016 ▸) using fragment **A** (Fig. 7 ▸, *R* = any atom), yielded seven hits similar to the title mol­ecule, *viz*. FACPEI with *R* = benzyl (Abad *et al.*, 2020 ▸), 3-(2-oxo-3-phenyl­quinoxalin-1(2*H*)-yl)propyl (KOPKAF; Abad *et al.*, 2024 ▸) and 2-(2-oxooxazolidin-3-yl)ethyl [monoclinic form (UREREP01; Daouda *et al.*, 2020 ▸) and ortho­rhom­bic form (UREREP; Daouda *et al.*, 2011 ▸)]. The last three are **B** (BZOQUX10; Oberti *et al.*, 1978 ▸), **C** (YEFDUK; Moreau *et al.*, 2012 ▸) and **D** (VAQNAE; Kumar *et al.*, 2012 ▸). The quinoxaline moiety is closest to planar in BZOQUX10 [dihedral angle between constituent planes = 1.13 (1)°], while in UREREP it is furthest from planar [dihedral angle between constituent planes = 3.34 (16)°]. These two compounds also exhibit the smallest [30.60 (1)°] and largest [38.72 (16)°] angles of inclination of the phenyl group. The other structures show inter­mediate values for both angles, except for YEFDUK and VAQNAE where this angle is less than 5° because the phenyl ring is part of a six- or five-membered ring fused to the nitro­gen-containing heterocycle.

## 5. Synthesis and crystallization

3-Phenyl­quinoxalin-2(1*H*)-one (1 g, 4.5 mmol), 3-bromo­prop-1-yne (0.96 mL, 9 mmol), and potassium carbonate (0.931 g, 6.75 mmol) with an amount of catalytic tetra-*n*-butyl­ammonium bromide (0.29 g, 0.9 mmol) were stirred in *N,N*-di­methyl­formamide (DMF) (20 mL) for 48 h (Fig. 1 ▸). The solution was filtered, and the solvent was removed under vacuum. Di­chloro­methane (20 mL) was added, and the solution was filtered. The residue was chromatographed on a silica gel column (hexa­ne/ethyl acetate: 9.5/0.5, as mobile phase) to give two fractions. The first fraction was purified by recrystallization in ethanol to afford colorless crystals with a yield of 28.3% (*O*-alkyl­ated isomer, title compound) while recrystallization of the second fraction gave a yellowish powder with a yield of 53.5% (*N*-alkyl­ated isomer).

***O*** **-alkyl­ated isomer**: Yield: 28.3%, m.p. = 370–372 K, ^1^H NMR (300 MHz, CDCl_3_) δ ppm: 2.55 (*t*, 1H, *CH*, *J* = 3Hz); 5.265 (*d*, 2H, O—C*H*_2_, *J* = 3Hz); 7.55–8.20 (*m*, 9H, C*H*_arom_); ^13^C NMR (75 MHz, CDCl_3_) δ ppm: 53.93 (O—*C*H_2_); 74.85 (*C*H); 78.57 (–*C*); 126.85, 127.22, 128.31, 129.06, 129.75, 129.82, 129.86 (*C*H_arom_); 136.77, 139.35, 139.49, 146.29 (*C*q); 154.11 (*C*q—O).

***N*** **-alkyl­ated isomer**: Yield 53.5%, m.p. = 385–387 K, ^1^H NMR (300 MHz, CDCl_3_) δ ppm: 2.35 (*t*, H, C*H*, *J* = 3Hz); 5.155 (*d*, 2H, N—C*H*_2_, *J* = 3Hz); 7.41–8.36 (*m*, 9H, C*H*_arom_); ^13^C NMR (75 MHz, CDCl_3_) δ ppm: 31.69 (N—*C*H_2_); 73.19 (*C*H); 76.96 (–*C*); 114.07, 124.15, 128.13, 129.61, 130.45, 130.53, 130.63 (*C*H_arom_); 131.87, 133.31, 135.78, 153.72 (*C*q); 153.98 (*C*=O).

## 6. Refinement

Crystal data, data collection and structure refinement details are summarized in Table 2 ▸. Hydrogen atoms were included as riding contributions in idealized positions with isotropic displacement parameters tied to those of the attached atoms.

## Supplementary Material

Crystal structure: contains datablock(s) global, I. DOI: 10.1107/S2056989024002585/vm2298sup1.cif

Structure factors: contains datablock(s) I. DOI: 10.1107/S2056989024002585/vm2298Isup2.hkl

Supporting information file. DOI: 10.1107/S2056989024002585/vm2298Isup3.cml

CCDC reference: 2142451

Additional supporting information:  crystallographic information; 3D view; checkCIF report

## Figures and Tables

**Table 1:** Hydrogen-bond geometry (Å, °)

*D*—H⋯*A*	*D*—H	H⋯*A*	*D*⋯*A*	*D*—H⋯*A*
C11—H11⋯N2^i^	0.95	2.44	3.3164 (14)	153

Symmetry code: (i) 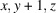 .

**Table 2:** Experimental details

Crystal data	
Chemical formula	C_17_H_12_N_2_O
*M*_r_	260.29
Crystal system, space group	Triclinic, *P* 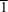
Temperature (K)	120
*a*, *b*, *c* (Å)	8.4614 (14), 9.0947 (15), 9.5360 (16)
α, β, γ (°)	87.739 (2), 72.963 (2), 69.028 (2)
*V* (Å^3^)	653.39 (19)
*Z*	2
Radiation type	Mo *K*α
μ (mm^−1^)	0.08
Crystal size (mm)	0.34 × 0.33 × 0.14

Data collection	
Diffractometer	Bruker SMART APEX CCD
Absorption correction	Multi-scan (*SADABS*; Krause *et al.*, 2015 ▸)
*T*_min_, *T*_max_	0.91, 0.99
No. of measured, independent and observed [*I* > 2σ(*I*)] reflections	12629, 3456, 2869
*R*_int_	0.026
(sin θ/λ)_max_ (Å^−1^)	0.684

Refinement	
*R*[*F* ^2^ > 2σ(*F* ^2^)], *wR*(*F* ^2^), *S*	0.044, 0.139, 1.17
No. of reflections	3456
No. of parameters	181
H-atom treatment	H-atom parameters constrained
Δρ_max_, Δρ_min_ (e Å^−3^)	0.42, −0.24

Computer programs: *APEX3* and *SAINT* (Bruker, 2016 ▸), *SHELXT* (Sheldrick, 2015*a* ▸), *SHELXL2019/1* (Sheldrick, 2015*a* ▸), *DIAMOND* (Brandenburg & Putz, 2012 ▸) and *SHELXTL* (Sheldrick, 2008 ▸).

## supplementary crystallographic information

### Crystal data

**Table:**

C_17_H_12_N_2_O	*Z* = 2
*M**_r_* = 260.29	*F*(000) = 272
Triclinic, *P*1	*D*_x_ = 1.323 Mg m^−^^3^
*a* = 8.4614 (14) Å	Mo *K*α radiation, λ = 0.71073 Å
*b* = 9.0947 (15) Å	Cell parameters from 6800 reflections
*c* = 9.5360 (16) Å	θ = 2.2–29.1°
α = 87.739 (2)°	µ = 0.08 mm^−^^1^
β = 72.963 (2)°	*T* = 120 K
γ = 69.028 (2)°	Thick plate, colourless
*V* = 653.39 (19) Å^3^	0.34 × 0.33 × 0.14 mm

### Data collection

**Table:**

Bruker SMART APEX CCD diffractometer	3456 independent reflections
Radiation source: fine-focus sealed tube	2869 reflections with *I* > 2σ(*I*)
Graphite monochromator	*R*_int_ = 0.026
Detector resolution: 8.3333 pixels mm^-1^	θ_max_ = 29.1°, θ_min_ = 2.2°
φ and ω scans	*h* = −11→11
Absorption correction: multi-scan (*SADABS*; Krause *et al.*, 2015)	*k* = −12→12
*T*_min_ = 0.91, *T*_max_ = 0.99	*l* = −13→12
12629 measured reflections	

### Refinement

**Table:**

Refinement on *F*^2^	Primary atom site location: dual
Least-squares matrix: full	Secondary atom site location: difference Fourier map
*R*[*F*^2^ > 2σ(*F*^2^)] = 0.044	Hydrogen site location: inferred from neighbouring sites
*wR*(*F*^2^) = 0.139	H-atom parameters constrained
*S* = 1.17	*w* = 1/[σ^2^(*F*_o_^2^) + (0.0919*P*)^2^] where *P* = (*F*_o_^2^ + 2*F*_c_^2^)/3
3456 reflections	(Δ/σ)_max_ < 0.001
181 parameters	Δρ_max_ = 0.42 e Å^−^^3^
0 restraints	Δρ_min_ = −0.24 e Å^−^^3^

### Special details

**Table:**

Experimental. The diffraction data were obtained from 3 sets of 400 frames, each of width 0.5° in ω, colllected at φ = 0.00, 90.00 and 180.00° and 2 sets of 800 frames, each of width 0.45° in φ, collected at ω = –30.00 and 210.00°. The scan time was 10 sec/frame.
Geometry. All esds (except the esd in the dihedral angle between two l.s. planes) are estimated using the full covariance matrix. The cell esds are taken into account individually in the estimation of esds in distances, angles and torsion angles; correlations between esds in cell parameters are only used when they are defined by crystal symmetry. An approximate (isotropic) treatment of cell esds is used for estimating esds involving l.s. planes.
Refinement. Refinement of F^2^ against ALL reflections. The weighted R-factor wR and goodness of fit S are based on F^2^, conventional R-factors R are based on F, with F set to zero for negative F^2^. The threshold expression of F^2^ > 2sigma(F^2^) is used only for calculating R-factors(gt) etc. and is not relevant to the choice of reflections for refinement. R-factors based on F^2^ are statistically about twice as large as those based on F, and R- factors based on ALL data will be even larger. H-atoms attached to carbon were placed in calculated positions (C—H = 0.95 - 1.00 Å). All were included as riding contributions with isotropic displacement parameters 1.2 - 1.5 times those of the attached atoms.

### Fractional atomic coordinates and isotropic or equivalent isotropic displacement parameters (Å^2^)

**Table:**

	*x*	*y*	*z*	*U*_iso_*/*U*_eq_	
O1	0.70891 (9)	0.56445 (8)	0.38257 (7)	0.02033 (18)	
N1	0.46180 (11)	0.60416 (9)	0.30849 (8)	0.01784 (19)	
N2	0.63397 (10)	0.29963 (9)	0.16411 (8)	0.01617 (19)	
C1	0.46804 (12)	0.39265 (11)	0.15776 (10)	0.0163 (2)	
C2	0.38348 (13)	0.33437 (11)	0.07839 (10)	0.0202 (2)	
H2	0.443192	0.233469	0.026117	0.024*	
C3	0.21438 (13)	0.42397 (12)	0.07686 (11)	0.0221 (2)	
H3	0.157226	0.384741	0.023461	0.027*	
C4	0.12524 (13)	0.57396 (12)	0.15423 (11)	0.0227 (2)	
H4	0.007481	0.633952	0.154015	0.027*	
C5	0.20634 (13)	0.63425 (11)	0.22970 (10)	0.0206 (2)	
H5	0.145802	0.736230	0.279815	0.025*	
C6	0.37993 (12)	0.54442 (11)	0.23271 (10)	0.0168 (2)	
C7	0.61911 (12)	0.51363 (11)	0.31114 (9)	0.0161 (2)	
C8	0.71038 (12)	0.35506 (10)	0.23996 (9)	0.0153 (2)	
C9	0.62072 (14)	0.72174 (11)	0.45585 (10)	0.0220 (2)	
H9A	0.664895	0.726667	0.540217	0.026*	
H9B	0.491675	0.744261	0.494533	0.026*	
C10	0.65155 (13)	0.84189 (11)	0.35597 (10)	0.0203 (2)	
C11	0.67695 (14)	0.94264 (12)	0.27984 (11)	0.0260 (2)	
H11	0.697268	1.023246	0.218929	0.031*	
C12	0.88656 (12)	0.24852 (11)	0.24864 (10)	0.0164 (2)	
C13	0.93639 (13)	0.24226 (12)	0.37693 (10)	0.0217 (2)	
H13	0.860641	0.313948	0.459583	0.026*	
C14	1.09717 (14)	0.13079 (12)	0.38327 (11)	0.0251 (2)	
H14	1.129576	0.125487	0.471192	0.030*	
C15	1.21016 (13)	0.02769 (12)	0.26279 (12)	0.0259 (2)	
H15	1.320346	−0.047128	0.267536	0.031*	
C16	1.16184 (13)	0.03400 (12)	0.13478 (11)	0.0243 (2)	
H16	1.239339	−0.036390	0.051699	0.029*	
C17	1.00061 (13)	0.14291 (11)	0.12807 (10)	0.0198 (2)	
H17	0.967552	0.145639	0.040722	0.024*	

### Atomic displacement parameters (Å^2^)

**Table:**

	*U*^11^	*U*^22^	*U*^33^	*U*^12^	*U*^13^	*U*^23^
O1	0.0248 (4)	0.0157 (3)	0.0240 (4)	−0.0084 (3)	−0.0109 (3)	0.0001 (3)
N1	0.0202 (4)	0.0157 (4)	0.0170 (4)	−0.0062 (3)	−0.0051 (3)	0.0012 (3)
N2	0.0171 (4)	0.0152 (4)	0.0165 (4)	−0.0063 (3)	−0.0050 (3)	0.0025 (3)
C1	0.0164 (4)	0.0157 (4)	0.0162 (4)	−0.0062 (3)	−0.0042 (3)	0.0038 (3)
C2	0.0216 (5)	0.0182 (5)	0.0222 (5)	−0.0084 (4)	−0.0072 (4)	0.0013 (4)
C3	0.0223 (5)	0.0253 (5)	0.0238 (5)	−0.0121 (4)	−0.0102 (4)	0.0049 (4)
C4	0.0170 (5)	0.0259 (5)	0.0234 (5)	−0.0058 (4)	−0.0069 (4)	0.0067 (4)
C5	0.0198 (5)	0.0182 (5)	0.0198 (4)	−0.0031 (4)	−0.0049 (4)	0.0025 (4)
C6	0.0181 (4)	0.0165 (4)	0.0151 (4)	−0.0062 (4)	−0.0041 (3)	0.0024 (3)
C7	0.0193 (5)	0.0155 (4)	0.0143 (4)	−0.0079 (4)	−0.0044 (3)	0.0024 (3)
C8	0.0171 (4)	0.0146 (4)	0.0144 (4)	−0.0066 (3)	−0.0041 (3)	0.0026 (3)
C9	0.0307 (5)	0.0184 (5)	0.0189 (4)	−0.0114 (4)	−0.0068 (4)	−0.0011 (4)
C10	0.0202 (5)	0.0182 (5)	0.0218 (4)	−0.0055 (4)	−0.0064 (4)	−0.0028 (4)
C11	0.0289 (6)	0.0208 (5)	0.0276 (5)	−0.0092 (4)	−0.0074 (4)	0.0020 (4)
C12	0.0170 (4)	0.0140 (4)	0.0203 (4)	−0.0072 (3)	−0.0066 (4)	0.0033 (3)
C13	0.0230 (5)	0.0217 (5)	0.0202 (4)	−0.0065 (4)	−0.0081 (4)	0.0016 (4)
C14	0.0277 (5)	0.0247 (5)	0.0286 (5)	−0.0095 (4)	−0.0171 (4)	0.0056 (4)
C15	0.0204 (5)	0.0205 (5)	0.0385 (6)	−0.0054 (4)	−0.0142 (4)	0.0049 (4)
C16	0.0203 (5)	0.0188 (5)	0.0292 (5)	−0.0025 (4)	−0.0064 (4)	−0.0018 (4)
C17	0.0210 (5)	0.0174 (4)	0.0218 (4)	−0.0065 (4)	−0.0081 (4)	0.0012 (4)

### Geometric parameters (Å, º)

**Table:**

O1—C7	1.3544 (11)	C8—C12	1.4832 (12)
O1—C9	1.4493 (12)	C9—C10	1.4648 (13)
N1—C7	1.2976 (12)	C9—H9A	0.9900
N1—C6	1.3751 (12)	C9—H9B	0.9900
N2—C8	1.3158 (11)	C10—C11	1.1874 (14)
N2—C1	1.3733 (11)	C11—H11	0.9500
C1—C2	1.4094 (12)	C12—C17	1.3961 (13)
C1—C6	1.4145 (13)	C12—C13	1.3991 (12)
C2—C3	1.3741 (13)	C13—C14	1.3922 (14)
C2—H2	0.9500	C13—H13	0.9500
C3—C4	1.4110 (15)	C14—C15	1.3831 (16)
C3—H3	0.9500	C14—H14	0.9500
C4—C5	1.3709 (14)	C15—C16	1.3895 (14)
C4—H4	0.9500	C15—H15	0.9500
C5—C6	1.4105 (13)	C16—C17	1.3876 (13)
C5—H5	0.9500	C16—H16	0.9500
C7—C8	1.4525 (13)	C17—H17	0.9500
			
C7—O1—C9	117.08 (7)	O1—C9—C10	111.63 (8)
C7—N1—C6	117.06 (8)	O1—C9—H9A	109.3
C8—N2—C1	118.73 (8)	C10—C9—H9A	109.3
N2—C1—C2	119.45 (8)	O1—C9—H9B	109.3
N2—C1—C6	120.70 (8)	C10—C9—H9B	109.3
C2—C1—C6	119.83 (8)	H9A—C9—H9B	108.0
C3—C2—C1	119.79 (9)	C11—C10—C9	177.21 (10)
C3—C2—H2	120.1	C10—C11—H11	180.0
C1—C2—H2	120.1	C17—C12—C13	119.09 (8)
C2—C3—C4	120.30 (9)	C17—C12—C8	118.53 (8)
C2—C3—H3	119.8	C13—C12—C8	122.21 (8)
C4—C3—H3	119.8	C14—C13—C12	119.89 (9)
C5—C4—C3	120.87 (9)	C14—C13—H13	120.1
C5—C4—H4	119.6	C12—C13—H13	120.1
C3—C4—H4	119.6	C15—C14—C13	120.63 (9)
C4—C5—C6	119.81 (9)	C15—C14—H14	119.7
C4—C5—H5	120.1	C13—C14—H14	119.7
C6—C5—H5	120.1	C14—C15—C16	119.73 (9)
N1—C6—C5	120.06 (9)	C14—C15—H15	120.1
N1—C6—C1	120.56 (8)	C16—C15—H15	120.1
C5—C6—C1	119.38 (9)	C17—C16—C15	120.12 (9)
N1—C7—O1	120.57 (8)	C17—C16—H16	119.9
N1—C7—C8	123.68 (8)	C15—C16—H16	119.9
O1—C7—C8	115.75 (8)	C16—C17—C12	120.54 (8)
N2—C8—C7	119.21 (8)	C16—C17—H17	119.7
N2—C8—C12	116.98 (8)	C12—C17—H17	119.7
C7—C8—C12	123.80 (8)		
			
C8—N2—C1—C2	−179.28 (8)	C1—N2—C8—C7	−1.53 (13)
C8—N2—C1—C6	−0.75 (13)	C1—N2—C8—C12	177.76 (7)
N2—C1—C2—C3	177.16 (8)	N1—C7—C8—N2	2.22 (14)
C6—C1—C2—C3	−1.38 (14)	O1—C7—C8—N2	−177.66 (7)
C1—C2—C3—C4	0.06 (14)	N1—C7—C8—C12	−177.02 (8)
C2—C3—C4—C5	1.26 (15)	O1—C7—C8—C12	3.10 (13)
C3—C4—C5—C6	−1.21 (15)	C7—O1—C9—C10	−87.00 (10)
C7—N1—C6—C5	177.76 (7)	N2—C8—C12—C17	33.33 (12)
C7—N1—C6—C1	−1.98 (14)	C7—C8—C12—C17	−147.42 (9)
C4—C5—C6—N1	−179.86 (8)	N2—C8—C12—C13	−141.76 (9)
C4—C5—C6—C1	−0.13 (14)	C7—C8—C12—C13	37.49 (13)
N2—C1—C6—N1	2.63 (14)	C17—C12—C13—C14	−0.54 (14)
C2—C1—C6—N1	−178.84 (8)	C8—C12—C13—C14	174.53 (8)
N2—C1—C6—C5	−177.10 (8)	C12—C13—C14—C15	1.21 (15)
C2—C1—C6—C5	1.42 (14)	C13—C14—C15—C16	−0.83 (16)
C6—N1—C7—O1	179.51 (7)	C14—C15—C16—C17	−0.23 (15)
C6—N1—C7—C8	−0.37 (14)	C15—C16—C17—C12	0.90 (14)
C9—O1—C7—N1	0.65 (13)	C13—C12—C17—C16	−0.51 (14)
C9—O1—C7—C8	−179.47 (7)	C8—C12—C17—C16	−175.76 (8)

### Hydrogen-bond geometry (Å, º)

**Table:**

*D*—H···*A*	*D*—H	H···*A*	*D*···*A*	*D*—H···*A*
C11—H11···N2^i^	0.95	2.44	3.3164 (14)	153

Symmetry code: (i) *x*, *y*+1, *z*.

## References

[bb1] Abad, N., Lgaz, H., Atioglu, Z., Akkurt, M., Mague, J. T., Ali, I. H., Chung, I.-M., Salghi, R., Essassi, E. M. & Ramli, Y. (2020). *J. Mol. Struct.* **1221**, 128727.

[bb2] Abad, N., Mague, J. T., Alsubari, A., Essassi, E. M., Alzahrani, A. Y. A. & Ramli, Y. (2024). *Acta Cryst.* E**80**, 300–304. 10.1107/S2056989024001518 PMC10915666 38456048

[bb3] Brandenburg, K. & Putz, H. (2012). *DIAMOND*. Crystal Impact GbR, Bonn, Germany.

[bb4] Bruker (2016). *APEX3* and *SAINT*, Bruker AXS, Madison, Wisconsin, USA.

[bb5] Daouda, B., Brelot, L., Doumbia, M. L., Essassi, E. M. & Ng, S. W. (2011). *Acta Cryst.* E**67**, o1235. 10.1107/S1600536811014632 PMC3089083 21754531

[bb6] Daouda, B., Doumbia, M. L., Hökelek, T., Zemmouri, F., Claude, K. A. L., Douira, A., Sebbar, N. K. & Essassi, E. M. (2020). *J. Mar. Chim. Heterocycl.* **19**, 55–69.

[bb7] Groom, C. R., Bruno, I. J., Lightfoot, M. P. & Ward, S. C. (2016). *Acta Cryst.* B**72**, 171–179. 10.1107/S2052520616003954 PMC4822653 27048719

[bb8] Krause, L., Herbst-Irmer, R., Sheldrick, G. M. & Stalke, D. (2015). *J. Appl. Cryst.* **48**, 3–10. 10.1107/S1600576714022985 PMC4453166 26089746

[bb9] Kumar, K. S., Adepu, R., Kapavarapu, R., Rambabu, D., Krishna, G. R., Reddy, C. M., Priya, K. K. K., Parsa, V. L. & Pal, M. (2012). *Tetrahedron Lett.* **53**, 1134–1138.

[bb10] Lgaz, H., ELaoufir, Y., Ramli, Y., Larouj, M., Zarrok, H., Salghi, R., Zarrouk, A., Elmidaoui, A., Guenbour, A., Essassi, E. M. & Oudda, H. (2015). *Der. Pharma Chem.* **7**, 36–45.

[bb12] Missioui, M., Said, M., Demirtaş, G., Mague, J. T., Al-Sulami, A., Al-Kaff, N. S. & Ramli, Y. (2022). *Arab. J. Chem.* **15**, 103595. 10.1016/j.arabjc.2021.103595 PMC8627592 34909067

[bb13] Moreau, S., Desplat, V., Savrimoutou, S., Massip, S., Deleris, G. & Guillon, J. (2012). *Compte Rend. Chim.* **15**, 753–757.

[bb14] Oberti, R., Coda, A., Incoccia, L. & Comin, F. (1978). *Acta Cryst.* B**34**, 1544–1548.

[bb15] Ramli, Y. & Essassi, E. M. (2015). *Adv. Chem. Res.* **27**, 109–160.

[bb11] Sheldrick, G. M. (2008). *Acta Cryst*. A**64**, 112–122. 10.1107/S0108767307043930 18156677

[bb16] Sheldrick, G. M. (2015*a*). *Acta Cryst.* A**71**, 3–8.

[bb20] Sheldrick, G. M. (2015*b*). *Acta Cryst.* C**71**, 3–8.

[bb17] Spackman, P. R., Turner, M. J., McKinnon, J. J., Wolff, S. K., Grimwood, D. J., Jayatilaka, D. & Spackman, M. A. (2021). *J. Appl. Cryst.* **54**, 1006–1011. 10.1107/S1600576721002910 PMC8202033 34188619

[bb18] Spek, A. L. (2020). *Acta Cryst.* E**76**, 1–11. 10.1107/S2056989019016244 PMC6944088 31921444

[bb19] Tan, S. L., Jotani, M. M. & Tiekink, E. R. T. (2019). *Acta Cryst.* E**75**, 308–318. 10.1107/S2056989019001129 PMC6399703 30867939

